# Resistance exercise improves working memory, but not set shifting in older adults at risk for diabetes

**DOI:** 10.1002/alz70861_108823

**Published:** 2025-12-23

**Authors:** Olivia R Ghosh‐Swaby, Jennifer Hanna Al‐Shaikh, Jane Thornton, Ali R. Khan, Daniel Palmer, Paul A Sheppard, Joyla A Furlano, Teresa Liu‐Ambrose, Timothy J Bussey, Lisa M Saksida, Lindsay S Nagamatsu

**Affiliations:** ^1^ Western University, London, ON Canada; ^2^ International Olympic Committee, Lausanne, Lausanne Switzerland; ^3^ Rodent Cognition Research and Innovation Core, London, ON Canada; ^4^ Queen's University, Kingston, ON Canada; ^5^ University of British Columbia, Vancouver, BC Canada; ^6^ Djavad Mowafaghian Centre for Brain Health, Vancouver, BC Canada; ^7^ Schulich School of Medicine and Dentistry, London, ON Canada; ^8^ Robarts Research Institute, London, ON Canada

## Abstract

**Background:**

Older adults at risk for type 2 diabetes (T2D) experience cognitive decline and brain health abnormalities, highlighting the need for early interventions. Resistance exercise training (RT) holds promise as a strategy to improve executive function, mitigate T2D risk factors, and support neuroplasticity, especially in aging. This study evaluated the effects of a 6‐month RT intervention compared to balance‐and‐tone (BAT; active control) exercise on cognitive outcomes, particularly set shifting, physiological measures, and biomarkers in older adults at risk for T2D.

**Methods:**

Sixty community‐dwelling adults (aged 60‐80 years) with risk factors for diabetes (body mass index ≥25, fasting glucose 6.1‐7.0 mmol/L, or CANRISK score ≥21) were randomized to twice‐weekly RT or BAT classes. Primary outcomes included set‐shifting performance and cognitive flexibility (Trail Making Test [TMT] Part B; TMT‐B minus TMT‐A). Secondary outcomes encompassed working memory (Digit Span Backward), global cognitive scores (Montreal Cognitive Assessment (MoCA), Alzheimer’s Disease Assessment Scale [ADAS‐Cog‐11]) and associative learning using a novel Bussey‐Saksida touchscreen cognitive task nearly identical to its rodent version. Additionally, Measures of mobility, fitness, glycemic control, and biomarkers were examined (brain derived neurotrophic factor, BDNF, insulin growth factor‐1, IGF‐1). An ANCOVA and repeated measures ANOVA was used to assess intervention effects on outcomes at endpoint and over time, respectively, with baseline performance and adherence as covariates (*p* <0.050 significant).

**Results:**

RT improved working memory (Digit Span Backward; *p* = 0.024) compared to BAT at endpoint, with a trend toward enhanced cognitive flexibility over time (TMT‐B minus TMT‐A; *p* = 0.049). No significant differences were observed in global cognitive scores (MoCA, ADAS‐Cog‐11). Bussey‐Saksida touchscreen tasks were adapted and validated for human use. Participants showed low accuracy on paired associative learning (54‐64% ± 4% accuracy), irrespective of training group. RT improved lower body strength over time (*p* =0.036), with no changes in glycemic levels. IGF‐1 levels decreased in the RT group (p = 0.037), with no changes in BDNF.

**Conclusions:**

Resistance exercise training offers targeted cognitive benefits in older adults at risk for diabetes, suggesting its potential as an intervention to address both T2D risk factors and cognitive decline. Further studies should explore long‐term impacts on brain structure and function.

